# A Method for generating marker-less gene deletions in multidrug-resistant *Acinetobacter baumannii*

**DOI:** 10.1186/1471-2180-13-158

**Published:** 2013-07-13

**Authors:** Ismawati Mohammad Amin, Grace E Richmond, Paromita Sen, Tse Hsien Koh, Laura JV Piddock, Kim Lee Chua

**Affiliations:** 1 Department of Biochemistry, Yong Loo Lin School of Medicine, National University of Singapore, 8 Medical Drive, Singapore 117597, Singapore; 2 Department of Pathology, Singapore General Hospital, Outram RoadSingapore169608; 3 School of Immunity and Infection, College of Medical and Dental Sciences, University of Birmingham, Edgbaston, Birmingham B15 2TT, UK

**Keywords:** *Acinetobacter baumannii*, Multidrug-resistant, AdeFGH, AdeIJK, RND pumps, Allelic replacement

## Abstract

**Background:**

*Acinetobacter baumannii* is an important nosocomial pathogen that has become increasingly resistant to multiple antibiotics. Genetic manipulation of MDR *A. baumannii* is useful especially for defining the contribution of each active efflux mechanism in multidrug resistance. Existing methods rely on the use of an antibiotic selection marker and are not suited for multiple gene deletions.

**Results:**

A tellurite-resistant (*sacB*^+^, *xylE*^+^) suicide vector, pMo130-Tel^R^, was created for deleting the *adeFGH* and *adeIJK* operons in two clinical MDR *A. baumannii,* DB and R2 from Singapore*.* Using a two-step selection, plasmid insertion recombinants (first-crossover) were selected for tellurite resistance and the deletion mutants (second-crossover) were then selected for loss of *sacB*. The DNA deletions were verified by PCR while loss of gene expression in the Δ*adeFGH*, Δ*adeIJK* and Δ*adeFGH*Δ*adeIJK* deletion mutants was confirmed using qRT-PCR. The contribution of AdeFGH and AdeIJK pumps to MDR was defined by comparing antimicrobial susceptibilities of the isogenic mutants and the parental strains. The deletion of *adeIJK* produced no more than eight-fold increase in susceptibility to nalidixic acid, tetracycline, minocycline, tigecycline, clindamycin, trimethoprim and chloramphenicol, while the deletion of *adeL*-*adeFGH* operon alone had no impact on antimicrobial susceptibility. Dye accumulation assays using H33342 revealed increased dye retention in all deletion mutants, except for the R2Δ*adeFGH* mutant, where a decrease was observed. Increased accumulation of ethidium bromide was observed in the parental strains and all pump deletion mutants in the presence of efflux inhibitors. The efflux pump deletion mutants in this study revealed that only the AdeIJK, but not the AdeFGH RND pump, contributes to antimicrobial resistance and dye accumulation in MDR *A. baumannii* DB and R2.

**Conclusions:**

The marker-less gene deletion method using pMo130-Tel^R^ is applicable for creating single and multiple gene deletions in MDR *A. baumannii*. The *adeFGH* and *adeIJK* operons were successfully deleted separately and together using this method and the impact of each efflux pump on antimicrobial resistance could be defined clearly.

## Background

*Acinetobacter baumannii*, a non-fementing Gram-negative cocco-bacillus, is a frequent cause of nosocomial bloodstream infections and is associated with considerable morbidity and mortality, especially among patients in intensive care or with burns [1]. *A. baumannii* has become increasingly resistant to multiple antibiotics, including imipenem and meropenem, the carbapenems of choice for treating multidrug resistant (MDR) *A. baumannii* infections. The incidence of carbapenem-resistant *A. baumannii* in the United States and Europe is around 54% and 16%, respectively, while the incidence in the Asia/Pacific rim is about 80% [2]. *A. baumannii* possesses a variety of intrinsic and acquired resistance determinants, including β-lactamases, class D oxacillinases, aminoglycoside-modifying enzymes, outer membrane proteins and active efflux systems [3]. Among its intrinsic resistance determinants, overexpression of the chromosomally encoded active efflux systems of the resistance-nodulation and division (RND) family, such as AdeABC, AdeFGH and AdeIJK pumps, are a mechanism of resistance to a number of antibiotics [4].

The impact of RND pumps to antibiotic resistance in *A. baumannii* has been demonstrated by inactivating the genes that encode the efflux pumps and the method for gene inactivation involves insertion of an antibiotic resistance gene to select mutants [5-7]. Studies using mutants in which RND efflux pump genes have been inactivated have suggested significant overlap in antibiotics that are substrates of the *A. baumannii* pumps. For instance, derivatives of the MDR clinical isolate BM4454 in which *adeABC* was inactivated had increased susceptibility to the same antibiotics (fluoroquinolones, chloramphenicol, tetracycline, tigecycline and erythromycin) as inactivation of *adeIJK* in the same isolate [6]. When both *adeABC* and *adeIJK* were inactivated in BM4454, increased susceptibility to ticarcillin, previously not observed in the Δ*adeABC* mutant or the Δ*adeIJK* mutant, was seen [6]. Furthermore, overexpression of a pump gene did not always result in an increase in the MIC of the same antibiotics that had increased activity in the pump inactivated mutants. For example, inactivation of *adeABC* in the MDR clinical isolate BM4454 did not affect its susceptibility to imipenem, amikacin and cotrimoxazole, but overexpressing *adeABC* in a non-MDR clinical isolate BM4587 increased the MIC of these antibiotics [4]. Therefore, it is possible that inactivation of a gene by inserting an antibiotic-resistance gene may affect the antimicrobial susceptibility of the pump gene-inactivated mutants, thus complicating the interpretation of the results.

To address this possibility and to define clearly the impact of each efflux pump on antibiotic resistance, we propose that genes encoding efflux pumps be deleted using a marker-less strategy first described by Hamad *et al* (2009) for *Burkholderia* spp. [8]. The suicide vector, pMo130 was modified to carry a tellurite resistance cassette, a non-antibiotic selection marker [9]. The *A. baumannii* isolates we have tested, including MDR isolates, were sensitive to tellurite and can be counter-selected in LB medium containing 30-60 mg/L tellurite. Gene deletion by allelic replacement was selected using a modification of the two-step process described by Hamad et al (2009) [8]. In this study, the *adeFGH* and *adeIJK* operons were deleted separately and together in two MDR *A. baumannii* strains, DB and R2. The *adeIJK* deletion mutant showed increased susceptibility to nalidixic acid, chloramphenicol, trimethoprim, tetracycline, tigecycline, minocycline and clindamycin, but the deletion of *adeL*-*adeFGH* operon had no impact on antimicrobial susceptibility in the two MDR isolates. Genetic and gene expression analyses revealed that the allelic replacement in both MDR strains had occurred. The marker-less gene deletion method we describe is robust and, unlike the creation of mutants by inserting an antibiotic resistance gene, is suitable for deleting multiple genes in MDR *A. baumannii*.

## Results

### Deletion of the A. *baumannii adeFGH* and *adeIJK* operons

**Figure 1:**
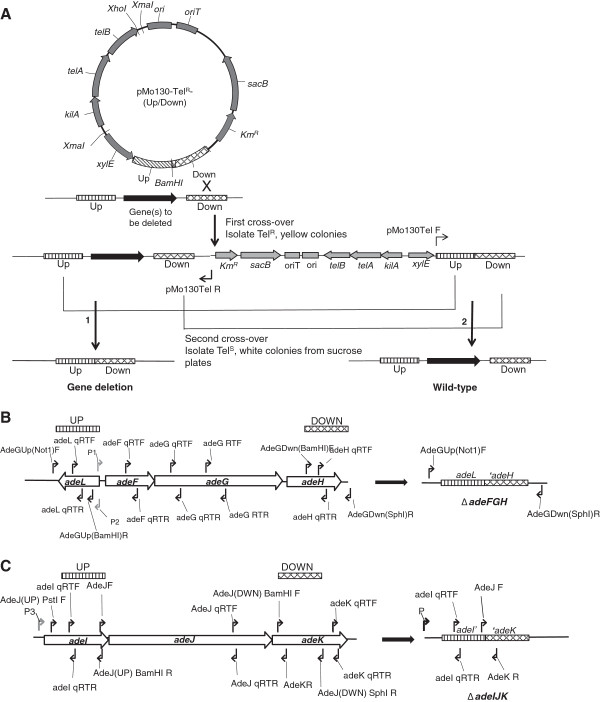
**Strategy for deleting *adeL-adeFGH *and *adeIJK *operons in MDR *A. baumannii *DB and R2.** Panel **A**, The upstream (UP) and downstream (DOWN) regions (approximately 1 kb) flanking the target genes was cloned into the suicide vector, pMo130-Tel^R^. pMo130-Tel^R^ was constructed by inserting a 3.26 kb *Xma*I-digested tellurite-resistance cassette from pwFRT-Tel^R^ into the *Xma*I site of pMo130. Recombinants obtained after first cross-over were selected for inheritance of tellurite-resistance and *xylE*^+^ (yellow colonies). These recombinants also do not produce any amplimers with the primer pair pMo130Tel F and pMo130Tel R. During the second cross-over, mutants with gene deletion (1) were selected for loss of *sacB* by passaging the first cross-over recombinants in media containing sucrose. The second cross-over could also yield parental genotype (2). Deletion of the *adeFGH* operon (Panel **B**) and the *adeIJK* operon (Panel **C**) showing the positions of the respective UP and DOWN fragments flanking each deletion (striped and hatched boxes, respectively). The locations of the PCR primers used for amplifying the UP and DOWN fragments and for qRT-PCR analysis of gene expression are indicated by black arrows while P1, P2 and P3 (grey arrows) are the locations of predicted promoters for *adeFGH* operon, *adeL*, and *adeIJK* operon, respectively.

To ensure reproducibility of the method, gene deletions were created for the *adeFGH* and *adeIJK* operons, separately and together, in two clinical MDR *A. baumannii* isolates, DB and R2. A suicide vector harboring a tellurite-resistance marker was first created by inserting a 3.26 kb *Xma*I-digested tellurite-resistance cassette from pwFRT-Tel^R^ into the *Xma*I site of pMo130 to give pMo130-Tel^R^[8,10]. In addition to the tellurite-resistance marker, pMo130-Tel^R^ also carries a kanamycin-resistance marker, the reporter gene *xylE* which converts pyrocathechol to a yellow-colored 2-hydroxymuconic semialdehyde, and a modified *sacB* gene [8]. Next, DNA fragments of approximately 1 kb upstream and 1 kb downstream of the target region to be deleted was ligated with linearized pMo130-TelR give pMo130-TelR-(Up/Down) (Figure 1A).

To construct the suicide plasmid for deletion of *adeFGH*, a 1 kb DNA fragment located upstream of *adeF* was amplified from R2 genomic DNA using the primer pair: AdeGUp(Not1)F and AdeGUp(BamHI)R (Figure 1B). The amplimer was digested using *Not*1 and *Bam*HI and inserted into pMo130-Tel^R^, creating pMo130-Tel^R^-*adeFGH*(UP). Next, another 1 kb fragment located downstream of *adeG* was amplified using the primer pair: AdeGDwn(BamHI)F and AdeGDwn(Sph1)R and cut with *Bam*HI and *Sph*I, and inserted into pMo130-Tel^R^-*adeFGH*(Up), thus creating pMo130-Tel^R^-*adeFGH*(Up/Down) (Figure 1B). The plasmid construct was first introduced in *E. coli* S17-1 and subsequently delivered into *A. baumannii* R2 and DB by biparental conjugation. *A. baumannii* transconjugants (first crossovers) were selected on LB agar containing 30 mg/L tellurite and 25 mg/L gentamicin. These tellurite-resistant colonies which carry genomic insertion of pMo130-Tel^R^-adeFGH (Up/Down) produced yellow colonies when sprayed with 0.45 M pyrocathechol and a 2 kb amplimer corresponding to the size of the ligated Up and Down DNA fragments with the primer pair: AdeGUp(Not1)F and AdeGDwn(Sph1)R, but did not produce any amplimer with the outward-facing primer pair: pMo130Tel F and pMo130Tel R (Figure 1A) (data not shown). *A. baumannii* R2 and DB harboring the inserted pMo130-Tel^R^-*adeFGH* (Up/Down) construct was cultured in LB broth containing 10% sucrose and passaged daily to select for deletion of *adeFGH* operon and loss of the *sacB* gene by a second cross-over and allelic replacement. Such bacteria, which were white when sprayed with 0.45 M pyrocathechol and were susceptible to 30 mg/L tellurite, usually appeared after the second passage. If the desired gene deletion had occurred, PCR of genomic DNA from these bacteria would produce only a 2 kb amplimer with the primer pair AdeGUp(Not1)F and AdeGDwn(Sph1)R. The same genomic DNA would not give any amplimer using the primer pair: AdeG RTF and AdeG RTR which annealed to the DNA that has been deleted (Figure 1B).

The suicide plasmid for deleting the *adeIJK* operon was constructed as described above but by first ligating the 1 kb UP fragment and a 0.9 kb DOWN fragment flanking the deletion before inserting into the pMo130-TelR vector (Figure 1C). The UP and DOWN fragments were amplified from R2 genomic DNA using the primer pairs, AdeJ(UP) PstI F and AdeJ(UP)BamHI R, and AdeJ(DWN)BamHI F and AdeJ(DWN)SphI R, respectively (Figure 1C and Additional file 1: Table S1). The UP and DOWN fragments were digested with *Bam*HI and ligated together in a 1:1 ratio. The ligated product was amplified using AdeJ(UP) PstI and AdeJ(DWN)SphI R to give a 1.9 kb amplimer which was then digested with *Pst*I and *Sph*I and ligated with pMo130-Tel^R^ linearized with *Pst*I and *Sph*I to give pMo130-Tel^R^-adeJ(Up/Down). The plasmid construct was introduced into *E. coli* S17-1 and used for the two-step selection for deletion of the *adeIJK* operon as described above.

### Verification of genomic deletions

**Figure 2:**
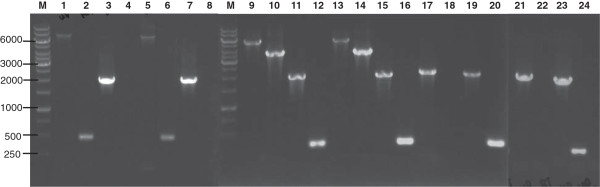
**Verification of genomic deletions by PCR of genomic DNA.** Amplimers using AdeGUp (NotI)F and AdeGDwn(SphI)R for DB (lane 1) and DBΔ*adeFGH* (lane 3) DNA; amplimers using AdeG RTF and AdeG RTR for DB (lane 2) and DBΔ*adeFGH* (lane 4) DNA; amplimers using AdeGUp (NotI)F and AdeGDwn(SphI)R for R2 (lane 5) and R2Δ*adeFGH* (lane 7) DNA; amplimers using AdeG RTF and AdeG RTR for R2 (lane 6) and R2Δ*adeFGH* (lane 8) DNA; amplimers using AdeJ(UP) PstI F and AdeJ(DWN) SphI R for R2 (lane 9), R2Δ*adeIJK* (lane 11), DB (lane 13) and DBΔ*adeIJK* (lane 15); amplimers using AdeJ F and AdeK R for R2 (lane 10), R2Δ*adeIJK* (lane 12), DB (lane 14) and DBΔ*adeIJK* (lane 16); DBΔ*adeFGH*Δ*adeIJK* DNA amplified using AdeGUp (NotI)F and AdeGDwn(SphI)R (lane 17), AdeG RTF and AdeG RTR (lane 18); AdeJ(UP) PstI F and AdeJ(DWN) SphI R (lane 19) and AdeJ F and AdeK R (lane 20); R2Δ*adeFGH*Δ*adeIJK* DNA amplified with AdeGUp (NotI)F and AdeGDwn(SphI)R (lane 21), AdeG RTF and AdeG RTR (lane 22), AdeJ(UP) PstI F and AdeJ(DWN) SphI R (lane 23) and AdeJ F and AdeK R (lane 24); M, 1 kb DNA ladder (GeneRuler™).

Genomic deletions of the *adeFGH* and *adeIJK* operons in the mutants were verified by comparing the PCR amplimers obtained from the parental isolates and corresponding pump gene deletion mutants. For the pump gene deletions, PCR using primers flanking the deletion produced a 2-kb amplimer corresponding to the UP and DOWN fragments (Figure 2, lanes 3, 7, 11, 15, 17, 19, 21 and 23) while a larger wild-type amplimer was obtained using genomic DNA from the parental isolates, R2 and DB (Figure 2, lanes 1, 5, 9 and 13). For the Δ*adeFGH* constructs, the deletion was also confirmed using PCR primers that annealed to the deleted region in *adeG*, whereby a 474 bp amplimer was obtained using genomic DNA from parental isolates (Figure 2, lanes 2 and 6), but no amplimer was obtained using genomic DNA from the Δ*adeFGH* deletion mutants (Figure 2, lanes 4, 8, 18 and 22). For the Δ*adeIJK* constructs, the deletion produced a 0.26-kb amplimer using the primers AdeJ F and AdeK R and genomic DNA from the Δ*adeIJK* mutants (Figure 2, lanes 12, 16, 20 and 24) and a longer 3.7-kb amplimer with genomic DNA from the wild-type parental isolates (Figure 2, lanes 10 and 14).

### Transcriptional analysis of the Δ*adeFGH* and Δ*adeIJK* deletion mutants

**Figure 3:**
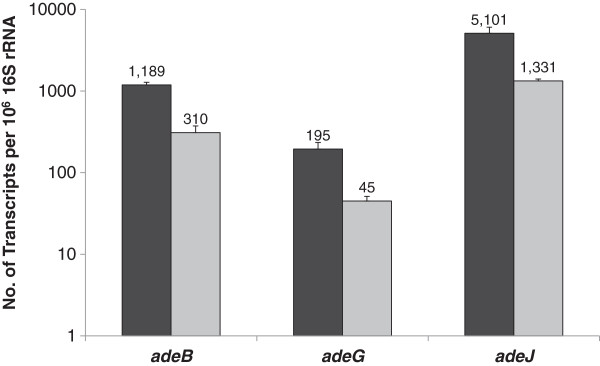
**Relative expression of *adeB*, *adeG *and *adeJ *in DB and R2 during mid-log phase.** RNA was extracted from mid-log phase bacteria (OD_600_ = 1.0) cultured in LB medium. The numbers of *adeB*, *adeG* and *adeJ* transcripts were each normalized to 16S rRNA transcripts. Black bars, DB; Light grey bars, R2.

RNA was extracted from parental strains and pump mutants cultured during mid-logarithmic growth in the absence of antibiotics. Analysis of the transcripts of the three major RND pumps in *A. baumannii* showed that the expression pattern of *adeB, adeG* and *adeJ* genes in both DB and R2 was similar (Figure 3). In the absence of any antibiotics, *adeIJK* was the most highly expressed pump while the expression of *adeFGH* was the lowest. All three pumps were also about 4-fold more highly expressed in DB as compared to R2 (Figure 3).

**Figure 4:**
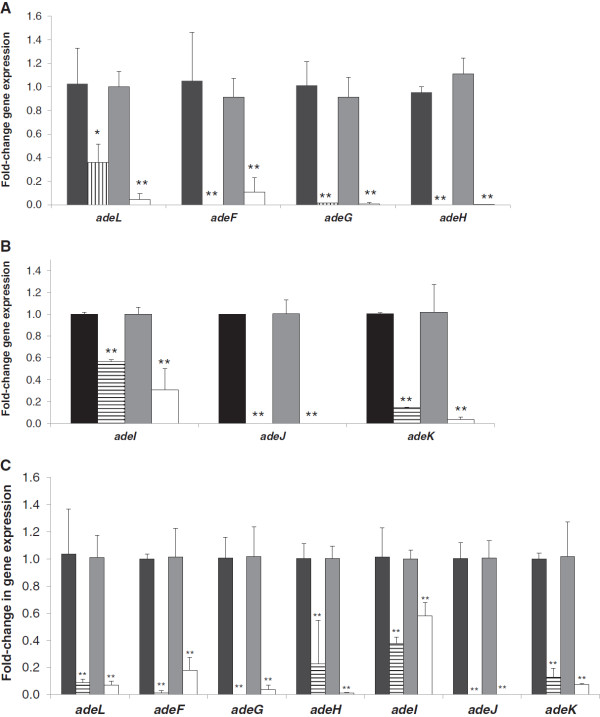
**Comparison of *adeL-adeFGH *operon and *adeIJK *operon expression in DB and R2 deletion mutants and parental strains.** Panel **A**, Fold-change in *adeL*, *adeF*, *adeG* and *adeH* expression in DB versus DBΔ*adeFGH*, R2 versus R2Δ*adeFGH*; Black bars, DB; grey bars, R2; vertical stripes, DBΔ*adeFGH;* white bars, R2Δ*adeFGH.* Panel **B**, Fold-change in *adeI*, *adeJ* and *adeK* expression in DB versus DBΔ*adeIJK*, and R2 versus R2Δ*adeIJK*; Black bars, DB; grey bars, R2; horizontal stripes, DBΔ*adeIJK;* white bars, R2Δ*adeIJK.* Panel **C**, Fold-change in *adeL*, *adeF*, *adeG, adeH*, *adeI, adeJ* and *adeK* expression in DB versus DBΔ*adeFGH*Δ*adeIJK*, and R2 versus R2Δ*adeFGH*Δ*adeIJK*. Black bars, DB; grey bars, R2; horizontal stripes, DBΔ*adeFGH*Δ*adeIJK;* white bars, R2Δ*adeFGH*Δ*adeIJK.* All differences in fold-change in gene expression between the parental strains and deletion mutants were significant (*, *p* < 0.05; **, *p* < 0.01).

To confirm that the gene deletions had abolished the expression of the efflux pumps, the levels of transcripts of each gene in the *adeFGH* and *adeIJK* operons were measured in the deletion mutants and compared with the corresponding transcript levels in the parental strains. Both the DBΔ*adeFGH* and R2Δ*adeFGH* mutants showed significant reduction (to ≤10%) in the transcript levels for *adeF, adeG* and *adeH* when compared to the parental strains (Figure 4A). Although detectable, the level of *adeL* transcription in these mutants was also significantly reduced when compared to the *adeL* transcripts in the parental strains. This was because the genomic deletion had included the putative *adeL* promoter. Inactivation of *adeG* in both DBΔ*adeFGH* and R2Δ*adeFGH* mutants was confirmed by the almost undetectable levels of *adeG* transcripts (Figure 4A).

Successful inactivation of *adeJ* was also similarly confirmed by the absence of *adeJ* transcripts in the DBΔ*adeIJK* and R2Δ*adeIJK* mutants (Figure 4B). A small quantity of *adeI* transcripts was udetectable in DBΔ*adeIJK* and R2Δ*adeIJK* mutants, albeit at 56% and 31% of wild-type levels, respectively. This was due to the location of the *adeI* qRT-PCR primers within the UP fragment, i.e. within the 5’ undeleted portion of the *adeI* gene (Figure 1C).

Next, we tested the feasibility of our marker-less deletion strategy for creating isogenic mutants carrying a combination of pump gene deletions. We applied this strategy to delete *adeIJK* in the DBΔ*adeFGH* and R2Δ*adeFGH* mutants to create DBΔ*adeFGH*Δ*adeIJK* and R2Δ*adeFGH*Δ*adeIJK* mutants, respectively. As expected, the DBΔ*adeFGH*Δ*adeIJK* and R2Δ*adeFGH*Δ*adeIJK* mutants showed significantly reduced expression of *adeL*, *adeF*, *adeG*, *adeH*, *adeJ* and *adeK* (Figure 4C). Expression of *adeI* in DBΔ*adeFGH*Δ*adeIJK* and R2Δ*adeFGH*Δ*adeIJK* mutants was reduced to 38% and 58% of DB and R2 levels, respectively.

### Antimicrobial susceptibility profiles of pump deletion mutants

The parental isolates, DB and R2, were MDR including to quinolones (nalidixic acid), fluoroquinolones (ciprofloxacin), chloramphenicol, tetracycline, carbapenems (meropenem and imipenem), β-lactams (piperacillin, oxacillin), cephalosporins (ceftazidime), macrolides (erythromycin), lincosamides (clindamycin), trimethoprim and aminoglycosides (gentamicin and kanamycin) (Table 1). Inactivation of the *adeIJK* in isolates DB and R2 resulted in at least a 4-fold increased susceptibility to nalidixic acid, chloramphenicol, clindamycin, tetracycline, minocycline and tigecycline, but had no effect on antimicrobial susceptibility to β-lactams (oxacillin and piperacillin), cephalosporins (ceftazidime), fluoroquinolones (ciprofloxacin), carbapenems (meropenem and imipenem), erythromycin and aminoglycosides (gentamicin and kanamycin). DBΔ*adeIJK* and R2Δ*adeIJK* mutants were also 8-fold more susceptible to trimethoprim when compared to the parental isolates.

**Table 1:** **Antimicrobial susceptibility of MDR*A. baumannii*DB, R2 and pump deletion mutants**

**Antibiotic**	**DB**	**DB∆*adeFGH***	**DB∆*adeIJK***	**DB∆*adeFGH*∆*adeIJK***	**R2**	**R2∆*adeFGH***	**R2∆*adeIJK***	**R2*∆adeFGH*∆*adeIJK***
Nalidixic acid	512	512	**128**	**128**	1024	1024	**256**	**256**
Ciprofloxacin	64	64	64	64	128	128	128	128
Chloramphenicol	64	64	**16**	**16**	128	128	**32**	**32**
Tetracycline	512	512	**128**	**128**	512	512	**128**	**128**
Minocycline	2	2	**0.25**	**0.25**	2	2	**0.5**	**0.5**
Tigecycline	1	1	**0.25**	**0.25**	1	1	**0.25**	**0.25**
Meropenem	128	128	128	128	64	64	64	64
Imipenem	32	32	32	32	64	64	64	64
Piperacillin	512	512	512	512	256	256	256	256
Oxacillin	> 1024	>1024	> 1024	>1024	1024	1024	1024	1024
Ceftazidime	256	128	256	256	256	128	512	512
Erythromycin	512	512	512	512	512	512	512	512
Clindamycin	128	128	**16**	**16**	128	128	**16**	**16**
Trimethoprim	128	128	**16**	**16**	128	128	**16**	**16**
Gentamicin	>1024	>1024	>1024	>1024	>1024	>1024	>1024	>1024
Kanamycin	>1024	>1024	>1024	>1024	>1024	>1024	>1024	>1024

MIC (mg/L).

Changes in MIC that are ≥ 4-fold are highlighted in bold.

Although *adeL* and the *adeFGH* operon were expressed in DB and R2, albeit at a lower level that *adeB* and *adeJ*, inactivation of *adeFGH* in both DB and R2 had minimal impact on the MDR phenotype of DB and R2 (Table 1). This is shown by the minimal change in antimicrobial susceptibility between the mutants that had only *adeFGH* inactivated (DBΔ*adeFGH* and R2Δ*adeFGH*) and both *adeFGH* and *adeIJK* operons inactivated (DBΔ*adeFGH*Δ*adeIJK* and R2Δ*adeFGH*Δ*adeIJK*) (Table 1). The DBΔ*adeFGH*Δ*adeIJK* and R2Δ*adeFGH*Δ*adeIJK* mutants had the same antimicrobial susceptibility as DBΔ*adeIJK* and R2Δ*adeIJK* mutants, respectively (Table 1).

### Growth of pump deletion mutants

The optical density at 600 nm measurements of liquid cultures of the parental strains and pump deletion mutants revealed no significant difference in growth kinetics (data not shown). Growth kinetics in the presence of sub-MIC concentrations of EIs were also carried out to simulate conditions in the H33342 accumulation assay (see below) and to ensure no inhibition of growth over a two-hour time period during the assay. These experiments showed that 30 mg/L CCCP and 50 mg/L PAβN did not restrict growth of R2 (data not shown). Viability of all strains was unaffected by H33342 concentrations of 2.5 μM, 5 μM and 10 μM (data not shown).

### Accumulation of H33342 by efflux pump gene deletion mutants

**Figure 5:**
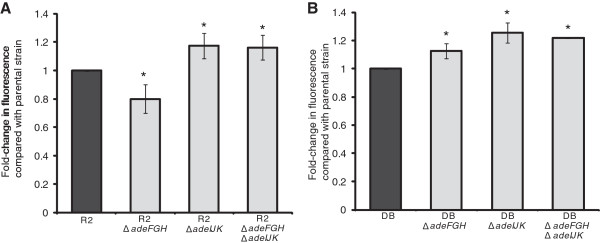
**Fold-change in fluorescence of H33342 at steady state levels of accumulation in efflux pump gene deletion mutants compared with the parental isolate.** Three separate experiments showed consistent results and the average fold change is shown. The standard deviation represents variation between three replicates of the assay. Asterisks indicate significant differences (*P* ≤ 0.05) in accumulation compared with the parental strain. Panel **A**, R2 and mutants. Panel **B**, DB and mutants.

Compared with the parental isolate, R2, there was a significant 0.8 fold change in the level of H33342 accumulated at steady state in R2Δ*adeFGH* (Figure 5A). Compared with the parental isolate, accumulation of H33342 was significantly increased in R2Δ*adeIJK* and R2Δ*adeFGH*Δ*adeIJK*, with a fold change of 1.18 and 1.16 respectively. The mutants created in isolate DB showed a different pattern of accumulation (Figure 5B). The level of H33342 accumulated at steady state was significantly higher in all three mutants, DBΔ*adeFGH,* DBΔ*adeIJK* and DBΔ*adeFGH*Δ*adeIJK,* compared with the parental strain, with fold-changes of 1.13, 1.26 and 1.22, respectively.

Addition of CCCP caused a significant increase in the steady state accumulation of H33342 by all strains (Table 2). In the R2 isolate and mutants, this increase was most pronounced in R2Δ*adeFGH*, with a fold increase of 1.46 observed (Table 2). The parental isolate showed a smaller fold increase of 1.31. R2Δ*adeIJK* and R2Δ*adeFGH*Δ*adeIJK* showed the smallest fold changes of 1.09 and 1.10, respectively. In the DB parent and mutant strain, the parental strain DB showed the highest fold increase of 1.51 after addition of CCCP, with the increase in DBΔ*adeFGH* slightly less, at 1.27 (Table 2). DBΔ*adeIJK* and DBΔ*adeFGH*Δ*adeIJK* again showed the smallest fold changes of 1.16 and 1.19, respectively. Addition of PAβN also caused a significant increase in accumulation in all strains (Table 2). This increase was of a similar fold in the parental strains, R2 and DB, and their mutants.

**Table 2:** Fold-change in fluorescence of H33342 at steady state level accumulation in the presence of EIs in efflux pump mutants and parental strains

**Bacterial strain**	**+CCCP**^**a**^	**+PAβN**^**b**^
DB	1.51 ± 0.04	1.29 ± 0.11
DBΔ*adeFGH*	1.27 ± 0.12	1.28 ± 0.03
DBΔ*adeIJK*	1.16 ± 0.06	1.24 ± 0.13
DBΔ*adeFGH*Δ*adeIJK*	1.19 ± 0.03	1.36 ± 0.07
R2	1.31 ± 0.12	1.27 ± 0.04
R2Δ*adeFGH*	1.46 ± 0.04	1.29 ± 0.03
R2Δ*adeIJK*	1.09 ± 0.01	1.29 ± 0.05
R2Δ*adeFGH*Δ*adeIJK*	1.10 ± 0.01	1.20 ± 0.10

Three separate experiments showed consistent results and representative examples are shown. The standard deviation represents variation between three biological replicates. All values shown are significant differences (P ≤ 0.05) in accumulation with addition of an EI relative to absence of EI.

^a^ fold-change compared to corresponding bacterial sample in the absence of CCCP.

^b^ fold-change compared to corresponding bacterial sample in the absence of PAβN.

### Accumulation of ethidium bromide by efflux pump gene deletion mutants

**Figure 6:**
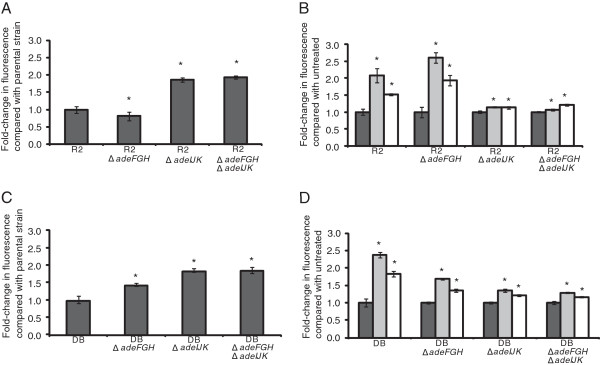
**Fold-change in fluorescence of ethidium bromide at steady state level of accumulation in efflux pump gene deletion mutants compared with the parental isolate.** Three separate experiments showed consistent results and representative examples are shown. Standard deviation represents variation between biological replicates. Asterisks indicate significant differences (*P* ≤ 0.05) in accumulation compared with the parental isolate or with addition of an EI. Panel **A**, Fold-change in level of ethidium bromide accumulated by R2 and mutants. Panel **B**, Fold-change in level of ethidium bromide accumulated by R2 and mutants with addition of EIs. Panel **C**, Fold-change in level of ethidium bromide accumulated by DB and mutants. Panel **D**, Fold-change in level of ethidium bromide accumulated by DB and mutants with addition of EIs. Dark grey, no EI; light grey, CCCP; white, PAβN.

It has been shown previously that H33342 and ethidium bromide are substrates of efflux pumps [11]. Therefore, accumulation of ethidium bromide was also measured. Compared with the parental isolate, the fold-change in the steady state levels of ethidium bromide accumulated in efflux pump mutants showed the same pattern as that produced with the H33342 accumulation assay, with levels in R2Δ*adeFGH* significantly lower than in parental isolate R2 (Figure 6A), and R2Δ*adeIJK* and R2Δ*adeFGH*Δ*adeIJK* accumulating significantly higher levels. Efflux pump mutants DBΔ*adeFGH,* DBΔ*adeIJK* and DBΔ*adeFGH*Δ*adeIJK* accumulated higher levels of ethidium bromide than the parental isolate, DB (Figure 6C). Addition of both CCCP and PAβN produced a significant increase in the level of ethidium bromide accumulated at steady state in both parental isolates and their mutants and the effect was similar to that seen with H33342.

## Discussion

The two-step deletion strategy we have described was used for creating unmarked deletions in the *adeFGH* and *adeIJK* efflux pump operons, separately and together, in two clinical MDR *A. baumannii* isolates. It is an improvement from the simple method for gene replacement *in A. baumannii* described by Aranda *et al* (2010) that uses an antibiotic resistance cassette [12]. To adapt the method first described for use in MDR *A. baumannii*, we introduced a tellurite resistance cassette into the pMo130 suicide vector created by Hamad *et al* (2009) to facilitate the selection of MDR *A. baumannii* transconjugants with the suicide plasmid inserted into the genome, i.e. first crossover products [8]. It was helpful to first ascertain the growth inhibitory concentration of tellurite for the parental *A. baumannii* strain so the number of transconjugants (first crossover) that are false positives can be minimized by using a suitable tellurite concentration. Passaging the first crossover recombinants in media containing sucrose provided the selection pressure for loss of the plasmid by a second crossover, leading to the formation of white colonies when sprayed with pyrocathechol.

The main advantage of this method, which does not use antibiotic selection for the gene deletion mutants, is its application for generating multiple gene deletions in a single strain as we have demonstrated by creating DBΔ*adeFGH*Δ*adeIJK* and R2Δ*adeFGH*Δ*adeIJK* mutants. This is particularly important because the majority of *A. baumannii* strains are MDR or extensively drug-resistant (XDR). Other than the MDR strains described in this study, we have also tested this method in a carbapenem-susceptible *A. baumannii* strain (data not shown). Un-marked deletion mutants are especially useful for ascertaining the contribution of each efflux pump to MDR as the presence of antibiotic resistance cassettes in the mutants may complicate the interpretation of antimicrobial susceptibility. We believe that the marker-less method would allow the impact of each efflux system on antimicrobial resistance to be clearly defined.

The contribution of efflux pumps to antibiotic resistance in *A. baumannii* has been demonstrated with mutants created by gene inactivation/deletion or by creating spontaneous efflux pump overexpressing mutants via selection on antibiotic gradients, but with some inconsistencies in antimicrobial susceptibilities depending on how the genes were inactivated [5]. For example, inactivation of *adeABC* in a clinical MDR isolate by insertion of a ticarcillin-resistance gene conferred increased susceptibility to aminoglycosides, β-lactams, fluoroquinolones, chloramphenicol, tetracycline, macrolides and trimethoprim [7]. However when *adeABC* was deleted and an apramycin resistance cassette was inserted in the same MDR isolate, the Δ*adeABC* mutant showed increased susceptibility to fluoroquinolones, chloramphenicol, tetracycline, tigecycline and macrolides but no change in susceptibility to aminoglycosides, trimethoprim and β-lactams [4,6]. We hypothesized that the antibiotic resistance gene used in the creation of pump gene mutants complicated the interpretation of antimicrobial susceptibility data and hence which agents were putative substrates of each *A. baumannii* efflux pump.

When *adeIJK* was inactivated using the marker-less method, the MDR isolates became more susceptible to nalidixic acid, chloramphenicol, clindamycin, tetracycline, minocycline, tigecycline and trimethoprim. It is interesting to note that the DBΔ*adeIJK* and R2Δ*adeIJK* mutants showed increased susceptibility to nalidixic acid without affecting susceptibility to ciprofloxacin, suggesting AdeIJK may be specific for quinolones but not fluoroquinolones. We also noted that, although the AdeIJK pump confers increased resistance to exactly the same antibiotics in both DB and R2, the host genotype had an influence on the magnitude of resistance to each antibiotic. The successful creation of *adeFGH* and *adeIJK* gene deletions, separately and together, in two MDR *A. baumannii* isolates demonstrates the robustness of the method and its application across different MDR *A. baumannii* isolates. The antibiotic substrates revealed with our mutants are in general agreement with those described by Damier-Piolle *et al* (2008) in which *adeIJK* was inactivated in an MDR isolate by gene deletion together with insertion of a kanamycin-resistance cassette [6]. However, in our study the DBΔ*adeIJK* and R2Δ*adeIJK* mutants were also more susceptible to trimethoprim, but not to β-lactams. It should be noted that differences between these studies may be due to the presence of different antibiotic resistance genes on the host genome, e.g. R2 had *bla*_*OXA-23*_ like*, bla*_*OXA-51*_ like genes*, bla*_*TEM*_*, bla*_*OXA*_ and *bla*_*ADC*_ that confer β-lactam resistance. The MICs of antibiotics for double mutants R2Δ*adeFGH*Δ*adeIJK* and DBΔ*adeFGH*Δ*adeIJK* were the same as for the corresponding single mutants R2Δ*adeIJK* and DBΔ*adeIJK*. This was expected, as a single deletion of *adeFGH* had minimal effect on MICs of antibiotics in either strain.

Deletion of *adeIJK* and of *adeFGH* in combination with *adeIJK* in both R2 and DB resulted in a significant increase in steady state accumulation levels of both H33342 and ethidium bromide. This infers reduced efflux in these strains, presumably as a consequence of the removal of the efflux pump AdeIJK. Addition of CCCP to Δ*adeIJK* and Δ*adeFGH*Δ*adeIJK* mutants of both R2 and DB significantly increased the steady state accumulation of H33342, suggesting that, despite lacking AdeIJK, these mutants still possess proton gradient dependent efflux activity as a result of another pump system. The addition of CCCP and PAβN had the same effect on the accumulation of ethidium bromide. However, the increase in accumulation observed in these mutants was not as high as that seen with the parental isolates and the *adeFGH* deletion mutants, supporting the previous finding that efflux is reduced in mutants lacking *adeIJK*.

In our study, the deletion of the *adeFGH* operon also removed the putative *adeL* promoter, resulting in reduced expression of *adeL*. However, both the inactivation of the *adeFGH* operon and reduced expression of *adeL* had very little impact on antimicrobial susceptibility when compared to the parental isolates which expressed both *adeL* and *adeFGH* operon. This was also true when the antimicrobial susceptibilities of DB and R2 mutants that had both the *adeIJK* and *adeFGH* operons deleted were compared with the DB and R2 mutants that had only the *adeIJK* operon inactivated. In all instances, inactivation of *adeFGH* had minimal impact on antimicrobial susceptibility when compared to isogenic isolates with functional AdeFGH, indicating that expression of *adeL* and *adeFGH* operon was not involved in the multidrug resistance of these clinical MDR isolates. These findings are different to those of Coyne *et al*, who showed that overexpressing *adeFGH* in an MDR strain lacking AdeABC and AdeIJK increased the MICs of several antibiotics including chloramphenicol, clindamycin, tetracycline, minocycline, tigecycline, norfloxacin, ciprofloxacin and cotrimoxazole [5]. In that study, the *adeFGH* operon was overexpressed in a spontaneous drug-resistant Δ*adeABC*Δ*adeIJK* mutant selected on norfloxacin and chloramphenicol gradient plates. The *adeFGH* operon was then deleted and a streptomycin-spectinomycin resistance cassette was also inserted to select for the deletion mutant. It is plausible that the process of selecting spontaneous drug-resistant mutants on chloramphenicol and norfloxacin gradients may have created gene duplication and amplification or a mutation in another efflux pump regulator was selected, especially since the inhibition of DNA gyrase by fluoroquinolones induces the SOS response [13]. It is also possible that under the experimental conditions whereby the *adeFGH* operon was induced and significantly overexpressed, an increase in resistance to chloramphenicol, trimethoprim and clindamycin may be observed. However, further studies are needed to determine the significance and relevance of such conditions to the clinical environment [14]. It was proposed that mutation in *adeL* results in overexpression of *adeFGH* operon and hence an increase in antibiotic resistance [5]. It is also possible that mutation in *adeL*, a LysR-type transcriptional regulator, may affect expression of another efflux pump gene/s or antibiotic resistance determinant. However, in the DBΔ*adeFGH* and R2Δ*adeFGH* mutants created in the present study, *adeL* expression was impaired yet there was minimal change in the MICs of antibiotics for the mutants when compared with the parental isolates. This ruled out the possibility that the MDR phenotype of DB and R2 might be due to a mutation in *adeL* which had an effect on the expression of another efflux pump(s) other than the *adeFGH* operon. Our data suggests that the activities of the AdeL transcriptional regulator and AdeFGH pump do not contribute to multidrug resistance in DB and R2.

Despite the minimal change in MICs of antibiotics compared with the parental isolate, R2Δ*adeFGH* showed a significant decrease in accumulation of both H33342 and ethidium bromide, inferring increased efflux in this strain. This may be due to increased expression of another efflux system in order to compensate for the loss of AdeFGH. This could also explain the lack of change in MIC seen with deletion of *adeFGH.* Previous work in *Salmonella enterica* serovar Typhimurium has shown that deletion of RND efflux pump genes can lead to compensatory altered expression of other efflux pump genes. For example, deletion of *acrB* in SL1344 resulted in a 7.9 fold increase in the expression of *acrF*[15]. An increase in accumulation of H33342 and ethidium bromide was seen in DBΔ*adeFGH*, inferring reduced efflux in this strain, however this difference did not translate into a change in MIC. Addition of CCCP and PAβN had a greater effect on accumulation of H33342 and ethidium bromide in this efflux pump mutant than in mutants lacking *adeIJK*. A greater fold change in accumulation was seen with both R2Δ*adeFGH* and DBΔ*adeFGH* than other efflux pump mutants, suggesting that efflux activity is higher in these mutants.

Using the marker-less deletion method, we have demonstrated that AdeFGH and AdeIJK are independent efflux pumps with no common antibiotic substrates. While both *adeFGH* and *adeIJK* operons are expressed in MDR *A. baumannii*, only the expression of *adeIJK* contributed to increased resistance to nalidixic acid, chloramphenicol, clindamycin, tetracycline, minocycline, tigecycline and trimethoprim. Expression of *adeFGH* was not the cause of resistance in the clinical isolates of MDR *A. baumannii*, DB and R2.

## Conclusions

The marker-less gene deletion method we have described is useful for creating gene deletions in MDR *A. baumannii*. Deletions of the *adeFGH* and *adeIJK* efflux pump operons, separately and together, were created in two clinical MDR *A. baumannii* isolates to demonstrate the robustness of the method. Even though both *adeFGH* and *adeIJK* operons are expressed in MDR *A. baumannii*, only the expression of *adeIJK* contributed to increased resistance to nalidixic acid, chloramphenicol, clindamycin, tetracycline, minocycline, tigecycline and trimethoprim. Expression of *adeFGH* was not the cause of resistance in the clinical isolates of MDR *A. baumannii*, DB and R2. This method allows the impact of each efflux system on antimicrobial resistance to be clearly defined.

## Methods

### Bacterial strains, plasmids and culture conditions

Bacterial strains and plasmids used in this study are listed in Table 3. *Acinetobacter baumannii* R2 (TTSH6013 654325/06) and DB (DB15354/07) were clinical isolates from a collection by the Network for Antimicrobial Resistance Surveillance, Singapore. According to the interim standard definitions for acquired resistance, both DB and R2 are classified as MDR as they are non-susceptible to ≥1 agent in ≥3 antimicrobial categories (aminoglycosides, fluoroquinolones, carbapenems, tetracycline, extended spectrum cephalosporins, folate pathway inhibitors) [17]. DB and R2 carry and express *bla*_OXA-23-like_ and *bla*_OXA-51-like_, do not carry *bla*_OXA-24-like_ and *bla*_OXA-58-like_ (data not shown). A*. baumannii* and *E. coli* were cultured under aerobic conditions at 37°C in Luria-Bertani Miller (LB) agar or LB broth (Becton Dickinson, Cockeysville, U.S.A.). Antibiotics used were at the following concentrations for *E. coli*: kanamycin, 10 mg/L; tellurite 6 mg/L; and for *A. baumannii*: tellurite, 30 mg/L.

**Table 3:** List of bacterial strains and plasmids used in this study

**Strain or plasmid**	**Relevant characteristics**	**Reference or source**
*A. baumannii* strains		
R2	Wild-type clinical MDR isolate TTSH6013 624325/06	Network for Antimicrobial Resistance Surveillance (Singapore)
DB	Wild-type clinical MDR isolate DB15354/07	Network for Antimicrobial Resistance Surveillance (Singapore)
R2Δ*adeFGH*	R2 with deletion in *adeFGH* operon	This study
R2Δ*adeIJK*	R2 with deletion in *adeIJK* operon	This study
R2Δ*adeFGH*Δ*adeIJK*	R2 with deletion in *adeFGH* and *adeIJK* operons	This study
DBΔ*adeFGH*	DB with deletion in *adeFGH* operon	This study
DBΔ*adeIJK*	DB with deletion in *adeIJK* operon	This study
DBΔ*adeFGH*Δ*adeIJK*	DB with deletion in *adeFGH* and *adeIJK* operons	This study
*E. coli* strains		
DH5α	F– Φ80*lac*ZΔM15 Δ(*lac*ZYA-*arg*F) U169 *rec*A1 *end*A1 *hsd*R17 *pho*A *sup*E44 λ– *thi*-1 *gyr*A96 *rel*A1	Invitrogen
S17-1	Genotype: *recA pro hsdR* RP4-2-Tc::Mu-Km::Tn7, Gm^S^	[16]
Plasmids		
pMo130	Suicide plasmid, *xylE*^+^, *sacB*^+^, Km^R^	[8]
pwFRT-Tel^R^	Donor of tellurite resistance cassette	[10]
pMo130-Tel^R^	pMo130 plasmid containing 3.26 kb *Xma*I-digested tellurite-resistance cassette from pwFRT-Tel^R^	This study
pMo130-Tel^R^-P8(UP/DWN)	pMo130-Tel^R^ containing a 1 kb UP fragment (promoterless *adeL*) and 1 kb DOWN fragment (3’ partial *adeH*)	This study
pMo130-Tel^R^-adeJ(Up/Down)	pMo130-Tel^R^ containing a 1 kb UP fragment (5’ partial *adeI*) and 0.9 kb DOWN fragment (3’ partial *adeK*)	This study

### DNA manipulations

Bacterial genomic DNA was extracted using a rapid procedure described by Pitcher *et al*[18]. Plasmid DNA was extracted using GeneAid Hi-Speed Plasmid Mini kit (GeneAid, Taiwan). Standard PCR amplifications were performed with Biotools DNA polymerase (Biotools, Spain). All primers used for PCR were synthesized by 1^st^ Base Singapore and are listed in Additional file 1: Table S1. Electrocompetent cells were prepared from 6 ml overnight bacterial culture according to the procedure described by Choi et al (2005) [19]. Electroporation was carried out by placing 100 μl electrocompetent cells and 3 μl plasmid DNA in a sterile cuvette (0.1 cm electrode gap, Bio-Rad) and pulsed at 1.8 V using settings for bacteria in a Bio-Rad MicroPulser.

The plasmid, pwFRT-Tel^R^, was digested with *XmaI* and the 3.265 kb fragment carrying the tellurite-resistance cassette was isolated and ligated with *Xma*I-linearized pMo130 to produce the suicide plasmid, pMo130-Tel^R^. The orientation of the tellurite-resistance cassette insert shown in Figure 1A was ascertained by digesting the plasmid with *Xho*1 and *Bam*HI which gave a 4.161 kb and a 5.231 kb band. An insertion of the tellurite-resistance cassette into pMo130-Tel^R^ in the opposite orientation would have produced two bands of 1.150 kb and 8.242 kb.

### Conjugative transfer

*E. coli* S17-1 donor strain harboring the respective pMo130-TelR-(Up/Down) constructs and the *A. baumannii* recipient strains were cultured overnight at 37°C in 2 ml LB (supplemented with kanamycin for the donor *E. coli* strain). Aliquots of 0.2 ml each of donor and recipient cells were added to a microfuge tube containing 1.2 ml of LB and washed twice with 2 ml LB each time. The cells were then suspended in 30 μl LB medium and added on to a sterile 0.45 μm cellulose nitrate filter paper (Sartorius Stedim, NY, U.S.A.) on LB agar and incubated at 30°C for 16 h. The cells were washed off from the filter by adding 0.4 ml of 0.9% NaCl. Aliquots of 0.1 ml were plated onto LB agar containing tellurite (30 mg/L) and gentamicin (25 mg/L) and incubated at 37°C for at least 16 h. Gentamicin was added for counter-selection against the donor cells.

### RNA analysis and quantitative real-time PCR (qRT-PCR)

RNA was extracted from mid-log phase bacteria prepared by inoculating 10 ml Luria-Bertani (LB) broth Miller (1^st^ BASE Pte Ltd, Singapore) with an overnight culture (1:50) and incubating at 37°C, with shaking at 120 rpm, until OD_600_ = 1.0. Triplicates of culture volumes containing two OD_600_ units (~ 2x10^9^ cells) were centrifuged at 3,000 g for 10 min to harvest the cells. The cells were lysed by adding 1 mL of TRIzol^®^ (Invitrogen, Carlsbad, CA) to the cell pellet and RNA was extracted according to the manufacturer’s protocol. Contaminating DNA was removed by treating the RNA sample with Ambion^®^ TURBO™ DNase (Invitrogen) and cDNA was synthesized using random hexamer primers and TaqMan^®^ Reverse Transcription Reagents (Invitrogen) according to the manufacturer’s protocol.

Primers for qRT-PCR of efflux pumps genes were designed using PrimerQuest (Integrated DNA Technologies, Coralville, IA) and are listed in Additional file 1: Table S2 (Supplementary files). qRT-PCR was performed using KAPA SYBR^®^ FAST Universal 2X qPCR Master Mix (Kapa Biosystems Inc., Woburn, MA) using 1X ROX (High) reference dye, 500 nm primers and ~10 ng cDNA in a total volume of 20 μL and the transcripts were detected using Applied Biosystems 7300 Real-Time PCR system (Applied Biosystems^®^, Carlsbad, CA). 16S rRNA was used for normalization of the qRT-PCR gene transcripts. qRT-PCR was performed twice for each of the triplicate RNA extracts. Data from each quantitative run was exported from the 7300 System software and analysed using 2^-∆∆Ct^ calculations [20].

### Antimicrobial susceptibility testing

Minimum inhibitory concentrations (MICs) of antibiotics were determined using the agar doubling dilution method according to BSAC standard methodology [21]. MICs of imipenem and meropenem were determined by E-test (Biomerieux, Hampshire, UK).

### Measurement of growth kinetics

Bacterial strains were grown with aeration in LB broth at 37°C overnight. Bacterial cultures were diluted 1:100 in sterile Luria Bertani (LB) broth and 100 μl of this suspension was added to each well of a clear 96 well microtitre tray. Optical density (OD) at an absorbance of 600 nm was measured over 16 hours in a BMG FLUOstar Optima (BMG, UK) at 37°C. The BMG FLUOstar is sensitive to an OD600 of between 0.0 and 4.0 and reproducibility is ±0.010 for the OD range of 0.0-2.0 ( http://www.bmglabtech.com). Each experiment included three biological replicates and three technical replicates of each bacterial strain. Differences in generation times and final OD at 600 nm were calculated using a Student’s *t*-test. *P* values ≤0.05 were considered as significant.

For assessment of toxicity of EIs and H33342, bacterial strains were grown with aeration in LB broth at 37°C overnight. A 4% inoculum (120 μl in 3 ml) of bacterial culture was added to fresh LB broth. This suspension was incubated with aeration at 37°C until the culture reached an OD at 600 nm of 0.6 (= 10^8^ cfu/ml). Cells were harvested by centrifugation at 2200 *g* for 10 min at room temperature and resuspended in 3 ml sterile LB broth at room temperature. The OD at 600 nm of the suspension was measured and adjusted to 0.5 to standardize the number of bacterial cells in each culture and to simulate the conditions used in the H33342 accumulation assay. The bacterial suspension (196 μl) was added to each well of a clear 96 well microtitre tray, along with 4 μl of EI and 20 μl H33342 at the required concentrations (see Results). OD at an absorbance of 600 nm was measured over 16 hours in the BMG FLUOstar OPTIMA (BMG, UK) at 37°C. Each experiment included three biological replicates and three technical replicates of each bacterial strain. Differences in generation times and final OD at 600 nm were calculated using a Student’s *t*-test. *P* values ≤0.05 were considered as significant.

### H33342 bis-benzamide accumulation in efflux pump deletion mutants

Accumulation of H33342 was measured and data analysed as described previously [11]. The level at which maximum fluorescence was reached and remained unchanged within the time period of the assay was taken as the steady state accumulation level. The fold change in fluorescence of mutants compared to the parental clinical isolate in the presence and absence of efflux pump inhibitors (EI) was calculated. Student’s *t*-tests were carried out to compare the accumulation of H33342 by the mutant with the parental strain, R2; *P* values <0.05 were taken as significant. Each assay was repeated 3 times with 3 biological replicates.

### Ethidium bromide accumulation in efflux pump deletion mutants

Ethidium bromide assays were carried out in the same way as the H33342 accumulation assay, except that cultures were resuspended in 1 M sodium phosphate buffer with 5% glucose. A 1 mM ethidium bromide stock solution was prepared and 20 μl was injected to give a final concentration of 0.1 mM in the assay. Fluorescence was measured over 117 minutes at excitation and emission wavelengths of 530 nm and 600 nm, respectively, in a FLUOstar OPTIMA.

## Authors’ contributions

IA and PS jointly constructed the *adeFGH* and *adeIJK* deletion mutants and carried out the molecular genetic studies. GER performed the dye accumulation antimicrobial susceptibility assays. THK provided the MDR *A. baumannii* isolates, characterized the *bla*_*OXA*_ sequences in DB and R2. KLC conceived the study. LJP and KLC participated in the design and coordination and helped to draft the manuscript. All authors read and approved the final manuscript.

## Supplementary Material

Additional file 1: Table S1 Description of primers used for PCR and DNA sequencing. **Table S2.** List of primers used for quantitative real-time PCR. Click here for file
