# Supplementary material for: A Method for generating marker-less gene deletions in multidrug-resistant Acinetobacter baumannii
Source: BMC Microbiol. 2013 Jul 13;13:158. doi: 10.1186/1471-2180-13-158 (PMC3717142; doi:10.1186/1471-2180-13-158)
Supplement: Additional file 1: Table S1 — Description of primers used for PCR and DNA sequencing. Table S2. List of primers used for quantitative real-time PCR. [file 1471-2180-13-158-S1.docx]

**SUPPLEMENTARY FILE**

**Table 1S.** Description of primers used for PCR and DNA sequencing

| **Primer name** | **Sequence** | **Product** |
| --- | --- | --- |
| AdeGUp(Not1)F | TATGCGGCCGCATTAAGTTTTGAGCGTATAAGCTTC | 1 kb UP fragment |
| AdeGUp(BamHI)R | GCTGGATCCATGAGAGTATTCAACAAAGT |  |
| AdeGDwn(BamHI)F | TATGGATCCAAATATTGCTCGTAACAGAG | 1 kb DOWN fragment |
| AdeGDwn(SphI)R | TATGCATGCAAGACGTAACTGGTAAAGAT |  |
| AdeG RTF | ACTTGTTGCAGTATTCGTAC | 474 bp adeG product, absent in R2Δ*adeFGH* |
| AdeG RTR | TTAAGTGCAGTGTCACTCAT |  |
| ABori (HindIII)F | CCGTAAGCTTGATCGTAGAAATATCTATGA | 1. 3kb *Acinetobacter* origin of replication from pWH1266 |
| AB ori (XhoI)R | TATTCTCGAGAAAGTTATCAACAGCCTAGA |  |
| AdeJ(UP) PstI F | TATCTGCAGGAAGTAGGTGTTATTGTTGC | 1 kb UP fragment |
| AdeJ(UP) BamHI R | TCTGGATCCTAAGGTTTTGCTGATACTTC |  |
| AdeJ(DWN) BamHI F | TATGGATCCCGTGAAAACCGATATACCAC | 0.9 kb DOWN fragment |
| AdeJ(DWN)SphI R | TCTGCATGCCCACCAGACTTAAATAGATC |  |
| AdeJ F | GCGATAAAGTCATTGTTGATG | 3.7 kb product from R2 and DB DNA; 0.26 kb product from Δ*adeIJK* mutant |
| AdeK R | GTTCTGTGTAATCTGATATTGCTG |  |
| pMo130Tel F | TTTACCACGACCGCATTCTC | Primers specific to pMo130Tel^R^ DNA flanking the Up and Down insert; PCR product not present after single and double crossover |
| pMo130Tel R | AAATAGGCGTATCACGAGGC |  |

**Table 2S.** List of primers used for quantitative real-time PCR

| **Target gene** | **Primer name** | **Sequence (5’🡪3’)** |
| --- | --- | --- |
| adeL | adeL qRTF  adeL qRTR | ACACGTCCACGTACTGACACACTT  GCAACCGCTGCTTCACCGATTTAT |
| adeF | adeF qRTF  adeF qRTR | AGCCGCTAGAGCTGCTGTCCA  CCCACGGTCACTTCAGCTCGT |
| adeG | adeG qRTF  adeG qRTR | CAAACATGGCGCGCCTCA  TGCCGATGGCAAGCACCA |
| adeH | adeH qRTF  adeH qRTR | AGCGAGTTGGGTAAATGGTCGAGT  CTTGAGCAACGCCTGCTTTACGTT |
| adeB | adeB qRTF  adeB qRTR | TTA AAC CGG GCA CAG ATG TG  GTA AAC CTT GCT GAC GTA CAA CTT G |
| adeI | adeI qRTF  adeI qRTR | AAGCACAAGTTGCAGCAGCTAAGG  AACGGGTCAGTCTGGTTTGCAGTA |
| adeJ | adeJ qRTF  adeJ qRTR | CAT TCT CGG CGT TTG CTA CAG  GGT GCA GGT GTA CCT TGA ATG TTA |
| adeK | adeK qRTF  adeK qRTR | CGTGCCAACATTGGTGAGCGTTTA  AAGAACGCTGCGCATCAAGAACAG |
| 16s rRNA | 16S qRTF  16S qRTR | ATCTTCGGACCTTGCGCTAA  ATCCTCTCAGACCCGCTACAGA |
